# Application of Genetically Engineered Pigs in Biomedical Research

**DOI:** 10.3390/genes11060670

**Published:** 2020-06-19

**Authors:** Magdalena Hryhorowicz, Daniel Lipiński, Szymon Hryhorowicz, Agnieszka Nowak-Terpiłowska, Natalia Ryczek, Joanna Zeyland

**Affiliations:** 1 Department of Biochemistry and Biotechnology, Poznan University of Life Sciences, Dojazd 11, 60-632 Poznań, Poland; lipinskidaniel71@gmail.com (D.L.); nwk.agnieszka@gmail.com (A.N.-T.); nataliaryczek.nr@gmail.com (N.R.); joanna.zeyland@up.poznan.pl (J.Z.); 2 Institute of Human Genetics, Polish Academy of Sciences, Strzeszyńska 32, 60-479 Poznań, Poland; szymon.hryhorowicz@igcz.poznan.pl

**Keywords:** genetically modified pigs, genome modifications, transgenic pigs, genetic engineering, disease models, recombinant proteins, xenotransplantation

## Abstract

Progress in genetic engineering over the past few decades has made it possible to develop methods that have led to the production of transgenic animals. The development of transgenesis has created new directions in research and possibilities for its practical application. Generating transgenic animal species is not only aimed towards accelerating traditional breeding programs and improving animal health and the quality of animal products for consumption but can also be used in biomedicine. Animal studies are conducted to develop models used in gene function and regulation research and the genetic determinants of certain human diseases. Another direction of research, described in this review, focuses on the use of transgenic animals as a source of high-quality biopharmaceuticals, such as recombinant proteins. The further aspect discussed is the use of genetically modified animals as a source of cells, tissues, and organs for transplantation into human recipients, i.e., xenotransplantation. Numerous studies have shown that the pig (*Sus scrofa domestica*) is the most suitable species both as a research model for human diseases and as an optimal organ donor for xenotransplantation. Short pregnancy, short generation interval, and high litter size make the production of transgenic pigs less time-consuming in comparison with other livestock species This review describes genetically modified pigs used for biomedical research and the future challenges and perspectives for the use of the swine animal models.

## 1. Introduction

Pigs have been extensively used in biomedical research due to anatomical and physiological similarities to humans. Moreover, progress in gene editing platforms and construct delivery methods allow efficiently, targeted modifications of the porcine genome and significantly broadened the application of pig models in biopharming and biomedicine.

Target editing is possible through site-specific nucleases, of which the following are most commonly used: zinc finger nucleases (ZFNs), transcription activator-like effectors (TALEs), and nucleases from the CRISPR/Cas (clustered regularly interspaced short palindromic repeats/CRISPR associated) system. Introducing modifications in a specific site of the genome is possible due to cellular processes of repairing double-strand breaks induced by site-specific nucleases. Double-strand breaks may be repaired in two ways: by non-homologous end joining (NHEJ) or by homologous recombination (HR). Repair provided by NHEJ may lead to the formation of indel (insertion/deletion) mutations in the target site that may be used to inactivate selected genes. In turn, repairing double-strand breaks by HR using a donor template allows for the introduction or elimination of specific point mutations and the introduction of any foreign genes. Pigs can be modified to create porcine models of human disease [1], produce recombinant proteins [2], or provide tissue and organs for xenotransplantation [3].

## 2. Techniques for Producing Genetically Engineered Pigs

Currently, microinjection or somatic cell nuclear transfer (SCNT) is most commonly used to obtain genetically modified animals. The microinjection is the oldest method for producing transgenic animals [4,5]. This well-developed technology involves the injection of the DNA material into the male pronucleus, the RNA material into the cytoplasm, or proteins into cytoplasm or pronucleus. Because of the difficulty in recognizing the male pronucleus, often, microinjection is simply made into one pronucleus. The efficiency of microinjection depends, among others, on the solution purity, its concentration, material form (DNA/RNA/protein), the length/size of the introduced structure (with increasing length/size the efficiency decreases), the skills of the operator, procedure, or embryo development stage. Due to the low efficiency of this procedure of about 10% in mice, 2%–3% in pig, 4% in rabbit, and less than 1% in cattle, researchers are introducing modifications to increase the number of successful microinjections and further transgenesis [6,7,8]. One of the introduced changes was the simultaneous injection of the transgene into both pronuclei. Increased transgene integration efficiency has been observed with the high invasiveness of the process resulting in high embryo mortality [9]. Subsequent tests were performed using DNA microinjection directly into the embryo cytoplasm. In this method, the problem of high invasiveness of injections was eliminated, while a problem arose with the integration of exogenous DNA and a further drastic decrease in the procedure efficiency [10]. This problem was eliminated by providing genetic material into the cytoplasm in the form of RNA. In this case, the stability of the material is a critical element. The third modification of DNA microinjection carried out by scientists was the delivery of the transgene into the cell nuclei of a two-blastomeric embryo. It was aimed at increasing the survival rate of the embryos. However, due to the higher probability of obtaining mosaic than transgenic animals, it is not commonly used in transgenesis [11]. Another method by which transgenic animals can be obtained is the use of transformed cells for somatic cloning. Interest in somatic cell nuclear transfer has been increasing since 1996 when the Dolly sheep was cloned for the first time [12]. This method, despite very low efficiency (0.5%–1.0% in livestock animals), allows for the use of a wide range of available approaches related to the cells’ genetic modification whose cell nuclei are later used for cloning. The choice of delivery method for exogenous DNA depends on its length, used cells, and transfection efficiency. After the identification of cells with appropriate modification, they should be brought to the G_0_ phase of the cell cycle. The cell nuclei of the modified cells are then transferred into the enucleated oocytes. The quality of oocytes and their age affect the cloning process. The whole method of obtaining transgenic animals by using transformed cells for somatic cloning has many advantages, but its main disadvantage is high fetal mortality resulting primarily from genetic defects [13]. A summary of the advantages and disadvantages of microinjection and SCNT is presented in Table 1.

## 3. Pig Models for Human Diseases

Genetically modified animals as research models for human diseases are a very important tool in searching for and developing new methods of therapy. A suitable model organism should be characterized by rapid growth, a high number of offspring, easy and inexpensive breeding, ability to be easily manipulated, and having a sequenced genome. Initially, only rodents were used as a model in biomedical research. Experiments on mice contributed to understanding the genetic background of numerous diseases. However, not every genetic disease induced in mice has the same clinical manifestation as in humans. Furthermore, the short life span, together with a higher metabolic rate, makes the analysis of some hereditary diseases challenging.

Currently, pigs are one of the most important large animal models for biomedical research. Many human diseases, such as cardiovascular diseases, obesity, and diabetes, have their counterparts in this species. The use of model organisms makes it possible to analyze diseases that occur naturally in animals with specific mutations and those deliberately induced. Introduced animal genome changes may reflect mutations occurring in people suffering from specific genetic disorders. Moreover, accurate and efficient genome editing can be used in the treatment of monogenic diseases. A slightly different direction of research is the use of genetically modified animal models in toxicological studies for testing drugs. Genetically modified pigs are used as model organisms in research into various diseases, including cardiovascular and neurodegenerative diseases, neoplasms, and diabetes.

### 3.1. Cystic Fibrosis

Cystic fibrosis (CF) is an autosomal recessive disorder manifested by bronchopulmonary failure and pancreatic enzyme insufficiency. CF is caused by a mutation in the gene responsible for the synthesis of the CFTR (cystic fibrosis transmembrane conductance regulator) chloride channel, altering the mucosal function in the respiratory epithelium, pancreatic ducts, intestines, and sweat glands. The most common cystic fibrosis-causing mutation is the deletion of a phenylalanine at amino acid position 508 (dF508). CF lung disease is the main cause of morbidity and mortality in CF patients. Porcine lungs share many anatomical and histological similarities with humans. It has been shown that pigs in which the *CFTR* gene was inactivated develop all symptoms of the disease occurring in humans, such as meconium ileus, defective chloride transport, pancreatic destruction, and focal biliary cirrhosis. This makes them a very good model species for this disease [14,15,16]. Cystic fibrosis is a monogenic disease, and the insertion of the functional *CFTR* gene into CF patient cells should theoretically restore the CFTR channel function. Therefore, pigs have also been used in gene therapy. The treatment with viral vectors successfully improved anion transport and inhibited bacterial growth [17,18]. Currently, the research focuses on improving the CF gene therapy with the use of the CRISPR/Cas9 system. These efforts are focused on increasing the delivery efficiency of CRISPR/Cas9 elements to target locus and obtaining sustained expression of the *CFTR* transgene [19,20]. It was demonstrated that precise integration of the human *CFTR* gene at a porcine safe harbor locus through CRISPR/Cas9-induced HDR-mediated knock-in allowed the achievement of persistent in vitro expression of the transgene in transduced cells. These results can help design effective gene therapy to treat CF patients [20].

### 3.2. Duchenne Muscular Dystrophy

Duchenne muscular dystrophy (DMD) is a progressive, monogenic, X-linked lethal disease characterized by degenerative changes in muscle fibers and the connective tissue. It involves the degeneration of subsequent muscles—skeletal, respiratory, and cardiac—and progressive muscular dystrophy. Muscular dystrophy is caused by a frameshift mutation in the *DMD* gene, which encodes dystrophin, a protein in muscle cells that connects the cytoskeleton with the cell membrane. The dystrophin gene contains 79 exons, with exons 3–7 and 45–55 being the most susceptible to mutations. *DMD* gene mutations are usually large deletions or duplications of one or several exons, as well as point mutations, leading to a change in the reading frame, the appearance of a premature stop codon, and failure to produce a stable protein. Muscular dystrophy is most often diagnosed in early childhood, and patients become wheelchair dependent by 12 years of age. Untreated boys die of cardiorespiratory complications around their 20 years. The rapid progress in gene editing gives hope for effective targeted therapies for DMD. Moreover, the use of an animal model can facilitate the development of personalized treatment approaches. Pigs with a DMD gene mutation (exon 52 deletion) develop human disease symptoms, such as lack of dystrophin in skeletal muscles, increased serum creatine kinase levels, progressive muscle dystrophy, and impaired mobility [21]. However, these animals died prematurely (up to 3 months old at most) what precluded natural breeding. The histological evaluation of skeletal muscles and diaphragm confirmed the presence of excessive fiber size variation, hypercontracted fibers, and segmentally necrotic fibers, resembled that of human DMD patients [21]. Moreover, proteome analysis of biceps femoris muscle was performed. An increased amount of muscle repair-related proteins and reduced amount of respiratory chain proteins was found in tissue from 3-month-old DMD pigs. This indicated severe disturbances in aerobic energy production and a decrease in functional muscle tissue [22]. As the deletion of exon 52 in the human *DMD* gene is a common cause of Duchenne muscular dystrophy, pigs can make an accurate research model for gene therapy. Another porcine DMD model is genetically modified miniature pigs with a mutation in exon 27 in the *DMD* gene obtained by the CRISPR/Cas9 system. In addition, these animals have shown symptoms of skeletal and heart muscle degeneration, characteristic of human patients with Duchenne muscular dystrophy. Reduced thickness of smooth muscle in the stomach and intestine was also observed in the pigs studied. However, founder pigs died of unreported causes [23]. Although mutations in exon 27 are not reported in human DMD patients, pigs with this deletion constitute another useful animal DMD model. Recently, Moretti et al. demonstrated the restoration of dystrophin by intramuscular injection of CRISPR/Cas9 components with the use of adeno-associated viral vectors in a pig model. In this study, pigs with DMD carrying a deletion of DMD exon 52 (d52DMD), resulting in a complete loss of dystrophin expression, were used. The restoration of dystrophin expression was possible due to the excision of exon 51 and the restoration of the DMD reading frame. The internally truncated d51-52DMD sufficed to improve skeletal muscle function, prevent malignant arrhythmias as well as prolonging lifespan of DMD pigs [24]. In the future, this strategy may prove useful in the clinical treatment of patients with d52DMD.

### 3.3. Alzheimer’s Disease

Alzheimer’s disease (AD) is an age-related, progressive neurodegenerative disorder characterized by memory dysfunction followed by cognitive decline and disorientation. AD accounts for 50%–80% of human dementia cases. Familial forms of AD are caused by autosomal mutations in the genes encoding presenilin 1 (*PSEN1*) and presenilin 2 (*PSEN2*) and amyloid precursor protein (APP). These mutations are associated with the accumulation of amyloid β (Aβ) peptide in senile plaques and phosphorylated tau protein in neurofibrillary tangles (NFTs), which leads to synaptic damage and neuronal dysfunction [25]. The first AD model with the use of transgenic pigs was generated in 2009 by Kragh et al. They produced Göttingen mini pigs that carried a randomly integrated construct containing the cDNA of the human *APP* gene with AD causing a dominant mutation known as the Swedish mutation (APPsw) and a human PDGFβ promoter fragment [26]. Although the transgene was specifically expressed in brain tissue at a high level, no AD phenotype was observed in mutant pigs. The same group also obtained Göttingen minipigs with the human *PSEN1* gene carrying the AD-causing Met146Ile mutation (*PSEN1* M146I) and driven by a cytomegalovirus (CMV)-enhanced human UbiC promoter. Pigs were generated with the use of a site-specific integration system—recombinase-mediated cassette exchange (RMCE) [27]. The PSEN1 M146I protein was expressed and tolerated well in the porcine brain, but also in this case, no symptoms of the AD disease were noticed. Therefore, this group generated double transgenic Göttingen minipigs with both APPsw and *PSEN1* M146I mutations. Such a solution allowed the increase in intraneuronal accumulation of Aβ [28]. In turn, another group obtained AD transgenic pigs using a retroviral multi-cistronic vector containing three AD-related human genes: APP, Tau, and *PSEN1*, with a total of six well-characterized mutations under the control of a fusion promoter: CMVE+ hPDGFβ promoter region. They confirmed that transgenes were expressed at high levels in brain tissue and demonstrated a two-fold increase in Aβ levels in the brains of transgenic pigs compared to wild-type [29].

### 3.4. Porcine Cancer Models

Cancer is a genetic disease involving uncontrolled, abnormal cell growth in the blood or solid organs resulting from acquired or inherited mutations. Pigs represent a useful animal for the development and validation of new medicines and procedures in human tumor models. There are many resemblances in cancer biology between pigs and humans. These animals can correctly mimic human tumors and show similar pharmacokinetic responses to humans. Adam et al. demonstrated that autologous transplantation of primary porcine cells transformed with retroviral oncogenic vectors caused tumorigenesis akin to those found in humans [30]. In turn, Schook et al. induced tumor formation in pigs by introducing random transgenes that encode Cre-dependent KRAS (Kirsten rat sarcoma viral oncogene homolog) G12D and TP53 (tumor protein 53) R167H oncogenic mutations (orthologous to human TP53 R175H) [31]. Moreover, Saalfrank et al. reported that porcine mesenchymal stem cells (MSCs) resemble human MSCs requiring disturbance of p53, KRAS, and MYC signaling pathways to become a fully transformed phenotype [32]. At present, pig models commonly used in cancer research include the TP53 knock-out model of osteosarcoma and APC (adenomatous polyposis coli) mutations model of familial adenomatous polyposis (FAP). *TP53* is a known tumor suppressor gene, and a germline mutation within this gene leads to Li–Fraumeni Syndrome, a rare, autosomal dominant disorder that predisposes carriers to cancer development. The first model of Li–Fraumeni Syndrome using genetically modified pigs has been described by Leuchs et al. They generated pigs carrying a latent *TP53* R167H mutation that can be activated by the Cre-lox recombinase system [33]. After several years of observation, it was noted that both pigs with homozygous *TP53* knock-out and pigs with heterozygous knock-out of *TP53* showed osteosarcoma development. The heterozygous knock-out caused the development of spontaneous osteosarcoma in older animals, while homozygous *TP53* knock-out resulted in multiple large osteosarcomas in 7 to 8-month-old pigs [32]. Moreover, Sieren and colleagues generated genetically modified Yucatan minipigs that carried the TP53 R167H mutation. Animals heterozygous for this mutant allele showed no tumorigenesis process, whereas homozygotes that reached sexual maturity developed lymphomas, osteogenic tumors, and renal tumors at varying rates. The tumor formations were validated by computed tomography, histopathological evaluation, and magnetic resonance imaging [34]. Familial adenomatous polyposis is an inherited disorder characterized by the development of numerous adenomatous polyps in the colon and rectum which greatly increases the risk of colorectal cancer. The mutations in the *APC* tumor-suppressor gene are responsible for FAP and may result in a hereditary predisposition to colorectal cancer. Flisikowska et al. generated gene-targeted cloned pigs with translational stop signals at codon 1311 in porcine APC (APC 1311), orthologous to common germline *APC* 1309 mutations in human FAP. Evaluation of one-year-old pigs carrying the *APC* 1311 mutation showed aberrant crypt foci and adenomatous polyps with low- to high-grade intraepithelial dysplasia, similar to tumor progression as in human FAP [35]. The APC 1311 pig model resulting in the development of polyposis in the colon and rectum can be useful in the diagnosis and therapy of colorectal cancer.

### 3.5. Cardiovascular Diseases

Cardiovascular diseases (CVDs) are the major cause of morbidity and mortality worldwide. CVD is a group of disorders of the heart and blood vessels that involve coronary heart disease (such as angina and myocardial infarction), deep vein thrombosis and pulmonary embolism, peripheral arterial disease, cerebrovascular disease, and rheumatic heart disease. The dominant cause of CVD is atherosclerosis, which is characterized by the narrowing of arteries due to the accumulation of lipid and plaque formation. The plaque buildup restricts blood flow, and plaque burst can entail blood clots. Similarities in heart anatomy and physiology, vessel size, blood parameters, coronary artery system anatomy, and lipoprotein metabolism make pigs a suitable model for the human cardiovascular system. Atherosclerosis starts with the buildup of serum low-density lipoprotein (LDL), and mutations in the LDL receptor (*LDLR*) gene may cause familial hypercholesterolemia (FH). A porcine FH model has been generated in Yucatan miniature pigs through recombinant adeno-associated virus-mediated targeted disruptions of the endogenous *LDLR* gene. LDLR+/− heterozygous pigs exhibited mild hypercholesterolemia, while LDLR−/− homozygotes animals were born with severe hypercholesterolemia and developed atherosclerotic lesions in the coronary arteries. These phenotypes were accelerated by high fat and high cholesterol diets [36]. The utilization of LDLR-deficient Yucatan minipigs in the preclinical evaluation of therapeutics was also demonstrated. LDLR+/− and LDLR−/− pigs were used to assess the ability of novel drug—bempedoic acid (BemA)—to reduce cholesterol biosynthesis. Long-term treatment with BemA decreased LDL cholesterol and attenuated aortic and coronary atherosclerosis in this FA model [37]. Moreover, a model of FA and atherosclerosis was created by using the Yucatan miniature pigs with liver-specific expression of a human proprotein convertase subtilisin/kexin type 9 (PCSK9) carrying the gain-of-function mutation D374Y. PCSK9 plays important functions in cholesterol homeostasis by reducing LDLR levels on the plasma membrane. Gain-of-function mutations in this protein cause increased levels of plasma LDL cholesterol, which in turn may result in more susceptibility to coronary heart disease. PCSK9 D374Y transgenic pigs exhibited decreased hepatic LDLR levels, severe hypercholesterolemia on high-fat, high-cholesterol diets, and atherosclerotic lesions [38]. It is also considered that hypertriglyceridemia is an independent risk factor for coronary heart disease, in which apolipoprotein (Apo)CIII is associated with plasma triglyceride levels. The hypertriglyceridemic ApoCIII transgenic miniature pig model was generated for the examination of the correlation between hyperlipidemia and atherosclerosis. Transgenic pigs expressing human ApoCIII exhibited increased plasma triglyceride levels with their delayed clearance and reduced lipoprotein lipase activity compared to non-transgenic controls [39].

### 3.6. Diabetes Mellitus

Diabetes mellitus (DM) is a group of metabolic disorders characterized by hyperglycemia (elevated levels of blood sugar over a prolonged period), which results from deficiency or ineffectiveness of insulin. DM may lead over time to cardiovascular disease, chronic kidney disease, damage to the nerves and eyes. There are two main types of diabetes mellitus, called type 1 and type 2. Type 1 DM, also referred to as juvenile diabetes or insulin-dependent diabetes mellitus, is caused by the pancreas’s failure to produce enough insulin. The most common is type 2 diabetes, which is characterized by insulin resistance (reduced tissue sensitivity to insulin) that may be combined with relative insulin deficiency. The anatomical and physiological resemblance to the human pancreas and islets makes pigs excellent animals for metabolic diseases modeling. Moreover, the structure of porcine and human insulin is also very similar (differs by only one amino acid). A transgenic pig model for type 2 DM was generated to evaluate the role of impaired glucose-dependent insulinotropic poly-peptide (GIP). The main function of incretin hormones GIP and glucagon-like peptide-1 (GLP1) is stimulated insulin secretion from pancreatic beta cells in a glucose-dependent manner. In type 2 DM, the insulinotropic action of GIP is impaired, which may suggest its association with early disease pathogenesis. The transgenic pigs expressing a dominant negative GIP receptor (GIPRdn) in pancreatic cells were produced by lentiviral vectors. A significant reduction in oral glucose tolerance due to delayed insulin secretion as well as in β-cell mass caused by diminished cell proliferation was observed in GIPRdn animals [40]. These observations resemble the characteristic features of human type 2 diabetes, which makes the porcine GIPRdn model useful for testing incretin-based therapeutic strategies. Further analyses revealed characteristic changes in plasma concentrations of seven amino acids (Phe, Orn, Val, xLeu, His, Arg, and Tyr) and specific lipids (sphingomyelins, diacylglycerols, and ether phospholipids) in the plasma of 5-month-old GIPRdn transgenic pigs that correlate significantly with β-cell mass [41]. These metabolites represent possible biomarkers for the early stages of prediabetes. Moreover, the porcine GIPRdn model has been used to test liraglutide, GLP1 receptor agonist, which improve glycemic control in type 2 diabetic patients. Ninety-day liraglutide treatment of adolescent transgenic pigs resulted in improved glycemic control and insulin sensitivity as well as reduction in body weight gain and food intake compared to placebo-treated animals. However, the use of liraglutide did not stimulate beta-cell proliferation in the endocrine pancreas [42]. Another type of diabetes, type 3 DM, is maturity-onset diabetes of the young (MODY3). MODY3 is a noninsulin-dependent type of diabetes with an autosomal dominant inheritance and is caused by mutations in the human hepatocyte nuclear factor 1 α (*HNF1α*) gene. Mutation in *HNF1α* gene leads to pancreatic β-cell dysfunction and impaired insulin secretion. A pig model for MODY3 was generated by expressing a mutant human *HNF1α* gene (HNF1α P291fsinsC) using intracytoplasmic sperm injection-mediated gene transfer and somatic cell nuclear transfer. The transgenic piglets exhibited the pathophysiological characteristics of diabetes, including high glucose level and reduced insulin secretion from the small and irregularly formed Langerhans Islets [43]. Furthermore, HNF1α P291fsinsC pigs revealed nodular lesions in the renal glomeruli, diabetic retinopathy, and cataract, complications similar to those in patients with DM [44]. Mutations in the insulin (INS) gene may result in permanent neonatal diabetes mellitus (PNDM) in humans. A PNDM large animal model was establish by generated pigs expressing a mutant porcine *INS* gene (INS C94Y), orthologous to human INS C96Y. Transgenic animals showed signs of PNDM, such as lower fasting insulin levels, decreased β-cell mass, reduced body weight, and cataract development. In addition, INS C94Y pigs exhibited significant β-cell impairment, including the reduction in insulin secretory granules and dilation of the endoplasmic reticulum [45]. The porcine INS C94Y model was further used to perform analysis of pathological changes in retinas and evaluation of the liver of transgenic pigs. The studies revealed several features of diabetic retinopathy, such as intraretinal microvascular abnormalities or central retinal edema [46]. Moreover, the multi-omics analysis of the liver demonstrated higher activities in amino acid metabolism, oxidation of fatty acids, gluconeogenesis, and ketogenesis, characteristic of insulin-deficient diabetes mellitus [47]. The genetically modified pig models for human diseases described in this review are summarized in Table 2.

## 4. Pigs as Bioreactors for Pharmaceutical Products

Human-derived proteins have long been used as therapeutics in the treatment of numerous diseases. However, their quantities are limited by the availability of human tissues. Thanks to the development of biotechnology and genetic engineering, modified animals can be used as “bioreactors” to produce recombinant proteins for pharmaceutical use. By using adequate regulatory sequences, promoters, the expression of transgenes can be directed to selected cells and organs. The therapeutic proteins can be obtained from milk, blood, urine, seminal plasma, egg white, or salivary gland that can be collected, purified, and used at an industrial scale. Moreover, it is possible to generate multi-transgenic animals that produce many biopharmaceuticals or vaccines in a single organism. The use of an animal platform allows for the relatively low-cost production of pharmacologically valuable preparations in high quantity and quality. The mammary gland is considered to be an excellent bioreactor system for pharmaceutical protein production. The advantage of milk is that it contains large amounts of foreign proteins that do not affect the animal’s health during lactation as well as the ease of product collection and purification. While cows are the best species for obtaining large amounts of pharmaceuticals in milk, the cost and time necessary to carry out successful transgenesis make rabbits, sheep, goats, and pigs more popular species. Although the pig is not a typical dairy animal, a lactating sow can give about 300 L of milk per year. Velander et al. generated transgenic pigs that synthesized human protein C in the mammary gland. Protein C plays an important role in human blood clotting, which makes it a potentially attractive drug. The collected milk contained 1 g/L of this protein [48]. Other recombinant human proteins involved in the coagulation process, such as factor VIII [49], factor IX [50,51], von Willebrand factor [52], were also successfully obtained in the porcine mammary gland. Furthermore, the line of transgenic pigs producing functional recombinant human erythropoietin in their milk was demonstrated. Erythropoietin regulates red blood cell production (erythropoiesis) in the bone marrow by binding to a specific membrane receptor and has been used in the treatment of anemia. This bioreactor system generates active recombinant human erythropoietin at concentrations of approximately 877.9 ± 92.8 IU/1 mL [53]. In turn, Lu et al. generated transgenic cloned pigs expressing large quantities of recombinant human lysozyme in milk. Lysozyme is a natural broad-spectrum antimicrobial enzyme which constitutes part of the innate immune system. The authors demonstrated that the highest concentration of recombinant human lysozyme with in vitro bioactivity was 2759.6 ± 265.0 mg/L [54]. Biopharmaceuticals can also be synthesized in pigs with the use of alternative systems, such as blood, urine, and semen. The blood of transgenic animals can be a source of human blood proteins, such as hemoglobin. Swanson et al. and Sharma et al. obtained transgenic pigs that produced recombinant human hemoglobin in their blood cells at a high level, with the ability to bind oxygen identical to that of human blood hemoglobin [55,56]. There remains, however, the issue of obtaining large amounts of animal-generated therapeutics easily and inexpensively, without killing the animal. Moreover, blood cannot store high levels of recombinant proteins for a long time, which are innately unstable, and bioactive proteins in the blood may affect the metabolism of the animals [57]. For this reason, research is being conducted into the production of recombinant proteins secreted into the urine or semen. The advantage of semen is that it is easily obtained and produced in high amounts in species such as pigs (boars can produce 200–300 mL of semen 2–3 times a week), while the advantage of urine is that proteins can be obtained from animals of both sexes throughout their lives. In addition, urine contains few proteins, which facilitates the purification of the protein product, and the urine-based systems pose a low risk to the animal’s health. However, the limitation of protein production in the bladder is low yield [58]. The recombinant pharmaceutical proteins produced from transgenic pigs are listed in Table 3.

## 5. Pig-to-Human Xenotransplantation

Genetically modified pigs can also be used as a source of cells, tissues, and organs for transplantation into human recipients. Despite the growing knowledge and ability to perform transplants, the shortage of organs means that the number of patients awaiting a transplant is constantly increasing. Xenotransplantation is any procedure involving the transplantation, implantation, or infusion of cells, tissues or animal donor organs, and also body fluids, cells, tissues, and human organs (or their fragments), which had ex vivo contact with animal cells, tissues, or organs into a human recipient. Organ xenotransplantation would give us an unlimited and predictable source of organs and enable careful planning of the surgery and preoperative drug treatment of the donor. The animal that best meets the criteria for xenotransplantation is the domestic pig (*Sus scrofa domestica*). Pig and human organs show great anatomical and physiological similarities. However, the significant phylogenetic distance results in serious immunological problems after transplantation. Despite major difficulties, the pig is currently the focus of all research aimed towards eliminating the problem of organ shortage for human transplantation in the future. Thus, the challenge now is to overcome interspecies differences that cause xenograft rejection by the human immune system. The solution, therefore, is to modify pigs in such a way that their organs are not rejected as belonging to another species. Advances in genetic engineering have brought scientists closer to obtaining modified animals that would be useful for pig to human transplants. A number of studies have reached the preclinical stage, using primates as model organisms.

### 5.1. Hyperacute Xenograft Rejection

Pig organs transplanted into human recipients are immediately rejected as a result of the so-called hyperacute immunological reaction. Xenograft rejection is mainly caused by the Gal antigen found on the donor’s cell surface, which is synthesized by the GGTA-1 enzyme. Humans lack both the Gal antigen and the GGTA-1 enzyme, but have xenoreactive antibodies directed against the porcine Gal antigen, which leads to the so-called enzymatic complement cascade in the recipient. The sequence of reactions results in a formation membrane attack complex, lysis, and destruction of the graft cells. The best possible solution to the problem of hyperacute rejection is to inactivate the gene encoding the GGTA-1 enzyme responsible for the formation of the Gal antigen. In 2001, the first heterozygous GGTA1 knock-out pigs were produced [59], and one year later, the first piglets with two knock-out alleles of the GGTA1 gene were born [60]. A series of GGTA1 knock-out pigs has also been generated by using ZFNs [61], TALENs [62], and the CRISPR/Cas9 system [63]. Moreover, other carbohydrate xenoantigens present on pig cells but absent in humans have been identified and include Neu5Gc antigen (N-glycolylneuraminic acid) catalyzed by cytidine monophosphate-N-acetylneuraminic acid hydroxylase (CMAH) and the SDa antigen produced by beta-1,4-N-acetyl-galactosaminyltransferase 2 (β4GALNT2). Pigs with GGTA1/CMAH/β4GalNT2 triple gene knock-out were generated using the CRISPR/Cas9 system. Cells from these genetically modified animals exhibited a reduced level of human IgM and IgG binding resulting in diminished porcine xenoantigenicity [64]. To prevent hyperacute rejection, it is possible to introduce human genes regulating the enzymatic complement cascade into the porcine genome. As the complement system may undergo spontaneous autoactivation and attack the body’s own cells, defense mechanisms have developed in the course of evolution. They regulate complement activity through a family of structurally and functionally similar proteins blocking complement activation and preventing the formation of a membrane attack complex (MAC). Introduction of human genes encoding complement inhibitors, such as CD55 (DAF, decay-accelerating factor), CD46 (MCP, membrane cofactor protein), CD59 (membrane inhibitor of reactive lysis), into the porcine genome may overcome xenogeneic hyperacute organ rejection [65]. It was demonstrated that the expression of human complement-regulatory proteins can prevent complement-mediated xenograft injury and prolong the survival time of the xenotransplant [66,67,68]. Studies have shown that the absence of GGTA1 and additional human CD55, CD59, or CD46 expression has greater survival rates than just GGTA1 knock-out [69,70]. Many genetically modified pigs with human complement inhibitors and other modifications important for xenotransplantation were also generated [71,72,73]. The modifications of the porcine genome described above largely resolved the problem of hyperacute rejection. However, xenogenic transplant becomes subject to less severe rejection mechanisms resulting from coagulation dysregulation, natural killer (NK) cells-mediated cytotoxicity, macrophage-mediated cytotoxicity as well as T-cell response.

### 5.2. Coagulation Dysregulation

The coagulative disorders result from incompatibilities between pig anticoagulants and human coagulation factors. Overcoming coagulation dysregulation in xenotransplantation will require the introduction of human gene encoding coagulation–regulatory proteins into the porcine genome, for example, thrombomodulin (TBM), endothelial cell protein C receptor (EPCR), tissue factor pathway inhibitor (TFPI), and ectonucleoside triphosphate diphosphohydrolase 1 (CD39). Thrombomodulin binds thrombin and functions as a cofactor for the activation of protein C, which is strongly anticoagulative. Porcine TBM binds human thrombin less strongly and cannot effectively activate protein C. It was demonstrated that expressing human TBM (hTBM) in porcine aortic endothelial cells (PAECs) suppresses prothrombinase activity and delays clotting time [71]. The endothelial protein C receptor enhances the activation of protein C and decreases proinflammatory cytokine synthesis. In vitro studies revealed the correlation between human EPCR (hEPCR) expression in PAECs and reduced human platelet aggregation [74]. A meta-analysis of multiple genetic modifications on pig lung xenotransplant showed that hEPCR was one of the modifications that had a positive effect on xenograft survival prolongation in the ex vivo organ perfusion model with human blood [75]. Further study demonstrated that kidneys from genetically-engineered pigs (carrying six modifications) functioned in baboons for 237 and 260 days. The authors suggested that prolonged survival time was associated, among others, with the expression of the human EPCR gene [76]. Tissue factor pathway inhibitor is the primary physiological regulator of the early stage of coagulation. TFPI binds to factor Xa, and then Xa/TFPI inhibits the procoagulant activity of the tissue factor (TF)/factor VIIa complex. It was demonstrated that the expression of human TFPI in PAECs can inhibit TF activity, suggesting potential for controlling the TF-dependent pathway of blood coagulation in xenotransplantation [77]. More recently, multi-modified pigs carrying human TFPI transgene were produced [76,78]. CD39 is an ectoenzyme that plays a key role in reducing platelet activation. CD39 converts adenosine triphosphate (ATP) and adenosine diphosphate (ADP) to adenosine monophosphate (AMP), which in turn is further degraded by ecto-5′-nucleotidase (CD73) to antithrombotic adenosine. Transgenic pigs with human CD39 (hCD39) gene were generated. The study showed that hCD39 expression protects against myocardial injury in a model of myocardial acute ischemia-reperfusion injury [79].

### 5.3. Inflammatory Response

Another approach to xenograft protection may be introducing a human gene that protects against the inflammatory response into the porcine genome. Transgenic pigs expressing antiapoptotic and anti-inflammatory proteins, such as human heme oxygenase-1 (HO-1) and human tumor necrosis factor-alpha-induced protein 3 (A20), were produced [80,81]. Porcine aortic endothelial cells derived from pigs carrying human A20 transgene were protected against TNF-α (tumor necrosis factor alpha)-mediated apoptosis and less susceptible to cell death induced by CD95 (Fas) ligands [81]. Similarly, overexpression of human HO-1 ensured prolonged porcine kidney survival in an ex vivo perfusion model with human blood and PAECs protection from TNF-α-mediated apoptosis [80]. Furthermore, pigs with a combined expression of human A20 and HO-1 on a GGTA1 knock-out background were generated. That transgenic approach alleviated rejection and ischemia-reperfusion damage during ex vivo kidney perfusion [82].

### 5.4. Cellular Xenograft Rejection

The cellular immune response is another barrier to xenotransplantation. Human NK cells can activate the endothelium and lyse porcine cells through direct NK cytotoxicity and by antibody-dependent cellular mechanisms. Direct NK cytotoxicity is regulated by activating and inhibitory receptor-ligand interactions. To prevent NK-mediated lysis through the inhibitory CD94/NKG2A receptor, pigs with human leukocyte antigens- E (HLA-E) were obtained [83,84]. The study showed that the expression of HLA-E in endothelial cells from transgenic pigs markedly reduces xenogeneic human NK responses. In addition, it was demonstrated that the introduction of the HLA-E gene into the porcine genome may also protect pig cells from macrophage-mediated cytotoxicity [85]. More recently, GGTA1 knock-out pigs with hCD46 and HLA-E/human β2-microglobulin transgenes were produced. The study showed that multiple genetically modified porcine hearts were protected from complement activation and myocardial natural killer cell infiltration in an ex vivo perfusion model with human blood [86]. Another approach to inhibit direct xenogeneic NK cytotoxicity is the elimination of porcine UL-16-binding protein 1 (ULBP1), which binds to NKG2D activating NK receptors. CRISPR technology was adapted to create genetically modified pigs with a disrupted UL16-binding protein 1 gene. In vitro studies confirmed that porcine aortic endothelial cells derived from ULBP1 knock-out pigs were less susceptible to NK-cells’ cytotoxic effects [87]. Macrophages also play an important role in xenograft rejection and can be activated by direct interactions between receptors present on their surface and donor endothelial antigens as well as by xenoreactive T lymphocytes. The binding CD47 antigen to macrophage surface signaling regulatory protein (SIRP-α) delivers a signal to prevent phagocytosis. However, the interaction between porcine CD47 and human SIRP-α does not supply the inhibitory effect on macrophages [88]. Therefore, the introduction of human CD47 (hCD47) into the porcine genome can overcome macrophage-mediated responses in xenotransplantation. The overexpression of hCD47 in porcine endothelial cells suppressed the phagocytic and cytotoxic activity of macrophages, decreased inflammatory cytokine (TNF-α, IL-6, IL-1β) secretion and inhibited the infiltration of human T cells [89]. The pigs with GGTA1 knock-out and hCD47 were obtained [90]. It was demonstrated that the expression of human CD47 markedly prolonged survival of donor porcine skin xenografts on baboons in the absence of immunosuppression [91]. Another challenge in xenotransplantation is the prevention of T cell-mediated rejection. T cells can be induced directly by swine leukocyte antigen (SLA) class I and class II on porcine antigen-presenting cells (APCs) or by swine donor peptides presented on recipient APCs. The main co-stimulatory signals regulating T cell function include CD40–CD154 and CD28–CD80/86 pathways. The cytotoxic T-lymphocyte antigen 4-immunoglobulin (CTLA4) can inhibit the CD28–CD80/86 co-stimulatory pathway. Therefore, the introduction of human CTLA4-Ig (hCTLA4-Ig) into the porcine genome may alleviate T cell response in xenografts. It was shown that neuronal expression of hCTLA4-Ig in pigs reduced human T lymphocyte proliferation [92]. Moreover, transgenic hCTLA4-Ig protein in pigs extended the survival time of porcine skin grafts in a xenogeneic rat transplantation model [93]. Another approach to inhibit T-cell immune response may be the deletion of swine leukocyte antigen class I. Reyes et al. created SLA class I knock-out pigs using gRNA and the Cas9 endonuclease. The obtained animals revealed decreased levels of CD4−CD8+ T cells in peripheral blood [94]. Recently, pigs carrying functional knock-outs of GGTA1, CMAH, B4GALNT2, and SLA class I with multi-transgenic background (hCD46, hCD55, hCD59, hHO1, hA20) were produced. In vitro study presented that the four-fold knock-out reduced the binding of human IgG and IgM to porcine kidney cells [95].

### 5.5. Porcine Endogenous Retroviruses

Beyond immune barriers in xenotransplantation, there is also concern about the risk of cross-species pathogens infection. The main problem constitutes porcine endogenous retroviruses (PERV), which are integrated into multiple locations in the pig genome. Utilizing the CRISPR/Cas9 technology gives great hopes for the complete elimination of the risk of PERV transmission. Niu et al. using the CRISPR/Cas9 system inactivated all 25 copies of functional PERVs in a porcine primary cell line and successfully generated healthy PERV-inactivated pigs via somatic cell nuclear transfer. What is more, no reinfection was observed in the obtained pigs [96].

### 5.6. Preclinical Studies in Xenotransplantation

Advances in genetic engineering and immunosuppressive therapies prolong organ survival time in preclinical pig-to-non-human primate (NHP) xenotransplantation models. The first xenotransplantation using pig hearts with eliminated Gal antigen into immunosuppressed baboons was performed in 2005. The longest surviving heterotopic graft functioned in the recipient for 179 days [97], in comparison to 4-6 hours of survival time with the use of wild-type pig hearts [98]. Introducing additional modifications extended the xenograft survival time even more. The longest survival was obtained for heterotopic cardiac xenotransplantation–up to 945 days. The authors used hearts derived from genetically multimodified pigs (GGTA1 knock-out, hCD46, hTBM) and chimeric 2C10R4 anti-CD40 antibody therapy [99]. Additional expression of hTBM in GGTA1 knock-out, hCD46 genetically modified pigs prevented early dysregulation of coagulation and prolonged the cardiac xenografts survival time [99,100]. Using the same genetic background, orthotopic heart xenotransplantation was performed, resulting in a maximum survival of 195 days [101]. However, xenograft survival time depends on the types of transplanted organs. In the case of kidneys in pig-to-NHP transplantation models, the longest survival of a life-sustaining xenograft was 499 days. GGTA1 knock-out pigs carrying hCD55 gene as well as immunosuppression with transient pan–T cell depletion and an anti-CD154–based regimen were used in the experiments. Moreover, the selection of recipients with low-titer anti-pig antibodies improved the long-term survival of pig-to-rhesus macaque renal xenotransplants [102]. The success of porcine liver and lung xenotransplantation remains limited, which is mainly associated with the occurrence of coagulation disorders [103]. The longest survival time for orthotopic liver xenografts (29 days) was achieved using GGTA1 knock-out pigs, exogenous human coagulation factors, and immunosuppression, including co-stimulation blockade [104]. In turn, Watanabe et al. demonstrated prolonged survival time of lung xenotransplants (14 days) from GGTA1 knock-out, hCD47/hCD55 donor pigs in immunosuppressed baboons [105]. The authors indicated the important role of hCD47 expression in reducing immunologic damages and extending lung graft survival in the pig-to-NHP model. However, additional genetic modifications of the porcine genome and immunosuppressive regimen strategy are necessary for the clinical application of xenotransplantation. Table 4 summarizes the most important genetic modifications of the porcine genome for xenotransplantation purposes.

## 6. Conclusions and Future Perspectives

**Figure 1:**
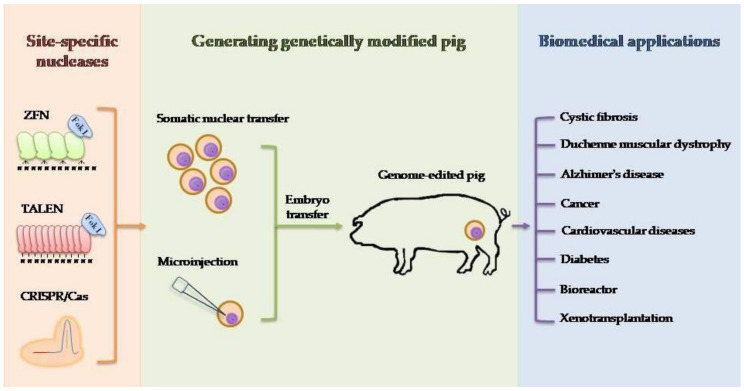
Schematic diagram of generation genetically engineered pig for biomedical purposes. The different site-specific nucleases (ZFN, TALEN, CRISPR/Cas9) used for genome editing and two techniques (somatic nuclear transfer and microinjection) to produce genetically modified pigs are shown. Biomedical applications for which genetically engineered pigs are generated include modeling human diseases, production of pharmaceutical proteins, and xenotransplantation.

The anatomical and physiological similarity between pigs and humans makes this species very interesting for biomedical research [110]. The rapid development of genetic engineering in recent years has allowed for precise and efficient modification of the animal genome using site-specific nucleases. The nuclease-mediated editing of the porcine genome, as well as potential applications of genetically modified pigs in biomedicine, are shown in Figure 1. Certainly, the driving force for development is the human mind and ideas that arise in it. One of the factors limiting the possibilities of using the potential of our ideas is the technical aspect. The transfer of new technologies, tested on smaller animal models, for example, is often limited and requires optimization for a large animal model. In the case of CRISPR/Cas9 technology, a lot of emphasis should be placed on the possibility of reducing the risk of so-called off-targets by improving this system. Paired nicking has the potential to reduce off-target activity in mice from 50–1000 times without compromising on-target performance [111]. Another strategy to limit the number of undesirable off-targets is to increase the specificity of the system. In this situation, one can focus on enhancing or improving Cas9 protein or sgRNA modifications. Protein Cas9 properties can be modified, or their lifespan can be changed [112,113]. For the future clinical success, it is also important to improve the efficiency of HR-mediated gene correction, especially in the situation of treating disease in which a template sequence is delivered to replace the mutated variant. Another important goal to achieve is the possibility of applying HR not only for dividing cells but also for cells in the post-mitotic stage. Hopes are placed in the fusion of the CRISPR/Cas9 technique and AAV (adeno-associated virus) as a donor template provider [114]. Considering the low immunogenicity of the AAV virus, the ability to transduce a wide spectrum of cells in terms of both type and developmental stage and strong limiting factor-capacity, it should be considered to minimize the CRISPR/Cas9 system or use more than one separate virus to simultaneously exploit the potential of both technologies. The use of other delivery systems, e.g., nanoparticles, is also worth considering [115].

Genetically modified pigs serve as an important large animal model for studying the genetic background of human diseases, testing novel drugs and therapy methods as well as developing models for gene therapy [116,117,118]. Pigs can be used as anatomical (e.g., endovascular), surgical, behavioral, and cytotoxic models. Ideas for new models of large animals are provided by the reality that shapes current demand. A lot has been done (pig model for influenza A infection), but still there is a need for pig models of other human viral diseases (hepatitis B; human immunodeficiency virus, HIV; severe acute respiratory syndrome coronavirus 2, SARS-CoV-2) [119]. HIV has been modeled in mice, filoviruses (Ebolavirus, Marburgvirus) have been modeled in small animals (i.e., mice, hamsters), but still, we need large models to investigate vaccines and antiviral drugs [120,121]. Transgenic pigs can also be a promising source of recombinant proteins used as pharmacological preparations. Actually, the possibility of using pigs for the production of biopharmaceuticals has been slowed in recent years. Some studies demonstrated that the pig mammary gland can be used as a complex recombinant protein source with appropriate post-translational modifications [122]. Despite the advantages of pig animal platform (natural secretion, correct posttranslational modifications, constant production), some ethical doubts are probably limiting the boost. Finally, the use of genetically engineered pigs for xenotransplantation is becoming an increasingly feasible alternative to standard allogeneic transplants and a potential solution to the problem of organ shortage. The combination of various multi-modified pigs and immunosuppressive therapies is required for overcoming immune rejection and effective xenotransplantation of different solid organs [123,124,125]. When it comes to treating end-stage organ failure, biomedical research could go a step further and try to create chimeric genetically modified pigs that would be carriers of human organs [126].

## Figures and Tables

**Table 1:** Summary of the most important advantages and disadvantages of the methods for obtaining genetically modified animals.

	Microinjection	SCNT
**Advantages**	increased efficiency of the transgene integration	precise transformation and selection of modified cells used in cloning
	the number of damaged zygotes do not exceed 10%	the obtained animals do not exhibit mosaicism
		modification is also revealed in germ cells—transgenic offspring
**Disadvantages**	low process efficiency (2%–3% in pigs)	very low efficiency
	the possibility of random integration of the transgene	early fetal mortality
	high process invasiveness	the possibility of genetic defects

**Table 2:** Selected genetically engineered pig models for human diseases.

Human Disease	Genetic Modification	Reference
Cystic fibrosis	targeted disruption of *CFTR* gene	[14,15,16]
Duchenne muscular dystrophy	targeted deletion of *DMD* exon 52 targeted knock-out of *DMD* gene	[21] [23]
Alzheimer’s disease	expression of human *APPsw* and *PSEN1* M146I genes expression of human *APP* (K670N/M671L, I716V, V717I), *Tau* (P301L), and *PSEN1* (M146V, L286P) genes	[28] [29]
Osteosarcoma	targeted knock-out of *TP53* gene targeted homozygous *TP53* R167H mutation	[32] [34]
Colorectal cancer	targeted heterozygous *APC* 1311 mutation	[35]
Cardiovascular Diseases	targeted disruption of *LDLR* gene expression of human *PCSK9* D374Y gene expression of human *ApoCIII* gene	[36] [38] [39]
Diabetes mellitus	expression of human *GIPRdn* gene expression of human *HNF1α* P291fsinsC gene expression of porcine *INS* C94Y gene	[40] [43] [45]

**Table 3:** Recombinant proteins produced from transgenic pigs for pharmaceutical use.

Protein	Production System	Yield	Reference
Human protein C	milk	up to 1 g/L	[48]
Human factor VIII	milk	up to 2.7 μg/mL	[49]
Human factor IX	milk	up to 0.25 mg/mL	[50]
Human von Willebrand factor	milk	mean 280 μg/mL	[52]
Human erythropoietin	milk	mean 877.9 IU/1 mL	[53]
Human lysozyme	milk	up to 2759.6 mg/L	[54]
Human hemoglobin	blood	up to 32 g/L	[56]

**Table 4:** Selected genetically engineered pigs for xenotransplantation.

Genetic Modification	Function	Reference
GGTA1 knock-out	deletion of Gal xenoantigen	[59,60]
CMAH knock-out	deletion of Neu5Gc xenoantigen	[106]
β4GALNT2 knock-out	deletion of SDa xenoantigen	[64]
expression of human CD55 gene	complement regulation	[107]
expression of human CD46 gene	complement regulation	[108]
expression of human CD59 gene	complement regulation	[65]
expression of human TBM gene	coagulation regulation	[109]
expression of human EPCR gene	coagulation regulation	[74]
expression of human TFPI gene	coagulation regulation	[77]
expression of human CD39 gene	coagulation regulation	[79]
expression of human HO-1 gene	anti-inflammatory/antiapoptotic	[80]
expression of human A20 gene	anti-inflammatory/antiapoptotic	[81]
expression of HLA-E	regulation of NK-cells-mediated responses	[83,84]
ULBP1 knock-out	regulation of NK-cells-mediated responses	[87]
expression of human CD47 gene	regulation of macrophage-mediated responses	[90]
expression of human CTLA4-Ig	regulation of T cells-mediated responses	[92]
SLA class I knock-out	regulation of T cells-mediated responses	[94]
PERV inactivation	xenozoonosis	[96]
